# Is being malnourished according to the ESPEN definition for malnutrition associated with clinically relevant outcome measures in geriatric outpatients?

**DOI:** 10.1007/s41999-018-0057-z

**Published:** 2018-05-03

**Authors:** N. M. van Rijssen, A. G. M. Rojer, M. C. Trappenburg, E. M. Reijnierse, C. G. M. Meskers, A. B. Maier, M. A. E. de van der Schueren

**Affiliations:** 1 Department of Internal Medicine, Section of Nutrition and Dietetics, VU University Medical Center, De Boelelaan 1118, Room ZH4A15, 1081 HV Amsterdam, The Netherlands; 2 Department of Internal Medicine, Section of Gerontology and Geriatrics, VU University Medical Center, Amsterdam, The Netherlands; 3 Department of Internal Medicine, Amstelland Hospital, Amstelveen, The Netherlands; 4 Department of Medicine and Aged Care, Royal Melbourne Hospital, University of Melbourne, Melbourne, Australia; 5 Department of Rehabilitation Medicine, VU University Medical Center, Amsterdam, The Netherlands; 6 Department of Human Movement Sciences, MOVE Research Institute Amsterdam, Vrije Universiteit, Amsterdam, The Netherlands; 7 Department of Nutrition, Sports and Health, Faculty of Health and Social Sciences, HAN University of Applied Sciences, Nijmegen, The Netherlands

**Keywords:** Malnutrition, Nutrition, Physical performance, Muscle strength, Depression, Aged

## Abstract

**Background and aim:**

A body of evidence is supporting the association between (the risk of) malnutrition in relation to physical performance, muscle strength, risk for depression and cognitive status in geriatric outpatients. Associations between being malnourished according to the newly proposed ESPEN definition for malnutrition and clinically relevant outcome measures of the aforementioned variables have not been confirmed yet. Therefore, the aim of this study was to examine the association between being malnourished according to the ESPEN definition and clinically relevant outcome measures in geriatric outpatients.

**Methods:**

Associations between malnutrition and handgrip strength (HGS, kg), short physical performance battery (SPPB-score, points), timed up and go test (TUG, seconds), and hospital anxiety and depression scale (HADS depression score, points), were analysed using linear regression. History of falls (falls, yes/no) and a low score on the Mini Mental-State Examination (MMSE-score ≤ 24 points) were analysed using logistic regression. All analyses were adjusted for age and gender.

**Results:**

A total of 185 geriatric outpatients (60% women) were included. The mean age was 82 (± 7.3) years. Being malnourished (8.2%) according to the ESPEN definition was significantly associated with a lower HGS (− 3.38 kg, *p* = 0.031), lower SPPB score (− 1.8 point, *p* = 0.025), higher TUG time (1.35 times higher time, *p* = 0.020) and higher HADS depression score (2.03 times higher score, *p* = 0.007). Being malnourished tended towards an association with falls (OR 3.84, *p* = 0.087). No significant association was found with low MMSE score (OR 2.61, *p* = 0.110).

**Conclusion:**

This study is the first to confirm the association between being malnourished, defined by the ESPEN definition and clinically relevant outcome measures in geriatric outpatients.

## Introduction

The European Society for Clinical Nutrition and Metabolism (ESPEN) proposed a new consensus definition for malnutrition in 2015. In this definition, fat-free mass index (FFMI) was introduced as an additional parameter to determine malnutrition, as FFMI provides important information on functional reserves and metabolic processes [1].

This new ESPEN definition seems to identify less malnourished patients compared to other tools [2, 3]. In geriatric (out)patients, malnutrition has previously been associated with poorer clinical outcomes, such as impaired muscle strength, worse physical performance, depression or worse cognitive status [4–7]. Therefore, the aim of the present study is to study whether the new ESPEN definition confirms this previously described associations, now that FFMI has been added as additional parameter to the definition.

## Materials and methods

### Study design

In this cross-sectional cohort study, 185 geriatric outpatients who were included who referred to the geriatric outpatient clinic of the Bronovo Hospital (The Hague, the Netherlands) between March 2011 and January 2012. All patients underwent a comprehensive geriatric assessment. No exclusion criteria were applied. This study was reviewed and approved by the institutional review board of the Leiden University Medical Centre (Leiden, the Netherlands). Data were obtained during routine care and the need for individual informed consent was waived by the ethical review board.

### Geriatric outpatient characteristics

Medical records were used to collect data on sex, age, polypharmacy (the use of five or more medicines) and multimorbidity (two or more of the following chronic diseases: hypertension, myocardial infarct, chronic obstructive pulmonary disease (COPD), Parkinson’s disease, diabetes mellitus, cancer, rheumatoid arthritis, and osteoarthritis. Unintentional weight loss (< 3 kg weight loss vs. ≥ 3 kg weight loss), current alcohol use (yes/no) and falls (yes/no) in the past 12 months were self-reported. Body mass index (BMI, kg/m^2^), fat-free mass (FFM, kg) and fat-free mass index (FFM/height^2^) were derived from direct segmental multi-frequency bioelectrical impedance analysis (DSM-BIA; In-Body 720; Biospace Co., Ltd, Seoul, Korea). Due to a protocol amendment BIA measurements were added at a later stage and performed in 135 out of 185 outpatients.

### ESPEN definition for malnutrition

After initial screening by a valid screening tool, the ESPEN definition comprises of two options to diagnose malnutrition [1]. The first option comprises a BMI below 18.5 kg/m^2^. The second option comprises unintentional weight loss (> 10% indefinite of time, or > 5% over the last 3 months), combined with either a low BMI (< 20 kg/m^2^ if < 70 years old or < 22 kg/m^2^ if ≥ 70 years old) or a low FFMI (female: < 15 kg/m^2^, male: < 17 kg/m^2^) [1]. Outpatients were diagnosed as malnourished (yes/no) if they fulfilled at least one of these options.

### Outcome measures

Muscle strength, physical performance, risk for depression, falls and cognitive status were considered as clinically relevant outcome measures. Handgrip strength (HGS in kg) [8] was used to measure muscle strength. The short physical performance battery (SPPB, 0–12 points) [9] and timed up and go (TUG, in seconds) [10] were used to measure physical performance. The Hospital Anxiety and Depression Scale (HADS depression score, 0–21 points, higher score indicating higher risk) [11], was used to measure the risk for depression. Falls in the past 12 months (y/n) indicated the presence of falls. The Mini Mental-State Examination Score (MMSE-score, 0–30, low score defined as < 24) [12] was used to measure cognitive status. All measurements were performed according to standard operating procedures.

### Statistical analysis

Continuous variables that were normally distributed are presented as mean and standard deviation. Skewed distributions are presented as median and interquartile range. The associations between being malnourished according to the ESPEN definition (independent variable) and HGS, SPPB-score, TUG and HADS depression score (dependent variables) were analysed using linear regression analysis. TUG and HADS were not normally distributed and were, therefore, log-transformed. After back transformation to normal, the interpretation should be interpreted as ‘times higher/lower compared to normal’ (proportional change).

Low MMSE score and falls (dependent variables) were analysed using logistic regression analysis. Age and sex were found to be confounders for the associations and thus included in the adjusted model.

Sensitivity analyses were performed excluding the 50 patients without a measurement of FFMI.

Data were analysed using the Statistical Package for the Social Sciences 22.0 (SPSS Inc., Chicago, Illinois, USA). *p*-values below 0.05 were considered statistically significant. *p*-values below 0.10 were considered as tending towards an association.

## Results

### Geriatric-outpatient characteristics

Table 1 shows the characteristics of the geriatric outpatients. Eight percent (*n* = 14) of patients were diagnosed malnourished: two (1.1%) had a BMI below 18.5 kg/m^2^, 11 (5.9%) had experienced unintentional weight loss in combination with a low BMI, and nine (4.9%) had experienced unintentional weight loss in combination with a low FFMI. Seven out of fourteen outpatients were malnourished according to more than one option of the ESPEN definition.

**Table 1:** Geriatric outpatient characteristics

	*N*	All
Age, years	185	82.0 (7.3)
Female, *n* (%)	185	111 (60.0)
Widowed, *n* (%)	183	78 (42.6)
Living independent, *n* (%)	145	59 (40.7)
Anthropometry		
Height, (cm)	177	167.1 (9.9)
Weight (kg)	173	71.9 (15.6)
BMI (kg/m^2^)	171	25.7 (4.4)
FFMI (kg/m^2^)	135	17.3 (2.8)
Male	55	18.7 (2.8)
Female	80	16.4 (2.4)
Parameters of health		
Unintentional weight loss, *n* (%)	185	24 (13)
Polypharmacy, *n* (%)^a^	180	110 (61.1)
Multimorbidity, *n* (%)^b^	177	67 (37.9)
Current alcohol use, *n* (%)	185	74 (40)
ESPEN definition for malnutrition		
Malnourished, *n* (%)	171	14 (8.2)
BMI < 18.5 (kg/m^2^)		2 (1.1)
Unintentional weight loss + low BMI		11 (5.9)
Unintentional weight loss + low FFMI		9 (4.9)
Clinically relevant outcome measures		
Handgrip strength, (kg)	181	26.1 (8.4)
Male	73	33.9 (6.1)
Female	108	20.8 (5.0)
SPPB score	179	7.0 (3.4)
TUG, seconds, median [IQR]	160	15.8 [11.8–21.8]
HADS depression score, median [IQR]	115	5.0 [3–9]
Falls in the past 12 months, *n* (%)	185	118 (63.8)
Low MMSE score, *n* (%)^c^	183	40 (21.9)

All numbers are presented as mean (SD) unless indicated otherwise

*BMI* body mass index, *FFMI* fat-free mass index, *ESPEN* European Society for Clinical Nutrition and Metabolism,* SPPB* short physical performance battery, *TUG* timed up and go test, *IQR* interquartile range, *HADS* Hospital and Anxiety Scale, *MMSE* mini-mental state examination

^a^Polypharmacy was defined as the use of five or more medicines

^b^Multimorbidity was defined as two or more of following chronic diseases: hypertension, myocardial infarct, COPD, cancer, diabetes mellitus, rheumatoid arthritis, osteoarthritis, Parkinson’s disease

^c^Low MMSE-score is defined as a MMSE-score < 24

### Associations between the ESPEN definition for malnutrition and outcome measures

Table 2 shows the results of the linear regression analyses for the association between being malnourished and HGS, SPPB score, TUG and HADS depression score.

**Table 2:** Associations between being malnourished according to the ESPEN definition and HGS, SPPB score, TUG and HADS depression score

	HGS (kg)			SPPB score			TUG^a, b^			HADS depression score^a,b^		
	*N* = 168			*N* = 165			*N* = 152			*N* = 115		
	*β*	95% CI	*p*	B	95% CI	*p*	B	95% CI	*P*	*β*	95% CI	*p*
Malnourished												
Crude model	− 4.035	− 8.896; 0.826	0.103	− 1.682	− 3.467; − 0.103	0.065	1.320	1.017; 1.714	**0.037**	1.948	1.158; 3.277	**0.012**
Model 1 (age and sex adjusted)	− 3.378	− 6.439; − 0.317	**0.031**	− 1.814	− 3.398; − 0.230	**0.025**	1.353	1.049; 1.745	**0.020**	2.026	1.124; 3.380	**0.007**

Interpretation: malnourished outpatients had a 3.38 kg lower (*p* = 0.031) handgrip strength compared to outpatients who were not malnourished

*p*-values below 0.05 are considered statistically significant.* p*-values below 0.10 are considered as tending towards an association

*CI* confidence interval, *HGS* handgrip strength, *SPPB* short physical performance battery, *TUG* timed up and go test, *HADS* Hospital Anxiety and Depression Scale

^a^Values were log transformed before analysis. After analysing, the log-transformed values were transformed back to normal

^b^Interpretation: malnourished outpatients had a 1.35 times higher timed up and go time compared to outpatients who were not malnourished

Being malnourished was significantly associated with lower HGS, lower SPPB score, a higher TUG time and a higher score on the HADS depression score, after adjustments for age and sex. Malnourished outpatients had a 3.38 kg lower HGS (*p* = 0.031), 1.8 points lower SPPB score (*p* = 0.025), a 1.35 times higher TUG time (*p* = 0.020), and a 2.03 times higher score on the HADS depression score (*p* = 0.007) compared to outpatients who were not malnourished.

Table 3 shows the results of the logistic regression analyses for the association between malnutrition, falls and low MMSE score. Malnutrition tended to be associated with falls; the odds on a fall was 3.84 higher (*p* = 0.087) compared to not being malnourished, adjusted for age and sex. No significant association between being malnourished and MMSE score was found.

**Table 3:** Associations between being malnourished according to the ESPEN definition and falls and low MMSE score

	Falls			Low MMSE score		
	*N* = 171			*N* = 169		
	OR	95% CI	*p*	OR	95% CI	*p*
Malnourished						
Crude model	3.916	0.847; 18.098	0.081	2.520	0.784; 8.097	0.121
Model 1 (age and sex adjusted)	3.841	0.822; 17.958	0.087	2.614	0.806; 8.482	0.110

Interpretation: malnourished outpatients have a 3.84 higher odds (p = 0.087) on a fall in the past 12 months compared to outpatients who were not malnourished (not significant)

Low MMSE score is defined as a MMSE-score < 24

*CI* confidence interval, *OR* odds ratio, *MMSE* mini-mental state examination

In outpatients with available FFMI (*n* = 135), the associations between the ESPEN definition for malnutrition and clinically relevant outcome were almost identical to the results in the total population. The association with SPPB score slightly attenuated from a 1.814 lower SPPB score (*p* = 0.025) in the total population to a 1.523 point lower SPPB score (*p* = 0.074) in the outpatients with FFMI available. The association with low MMSE score changed from a 2.614 higher odds (*p* = 0.110) on a low MMSE score in the total population into a 3.934 higher odds (*p* = 0.033) on a low MMSE score in outpatients with FFMI available.

## Discussion

This cross-sectional study is the first to describe the association between being malnourished based on the new ESPEN definition and clinically relevant outcome measures in geriatric outpatients. Being malnourished was associated with lower HGS, lower SPPB score, higher TUG time and higher HADS depression score. A trend was found between being malnourished and falls. No association was found between being malnourished and low MMSE score. The study confirms the low impact of having a low BMI alone on diagnosing malnutrition, and pleads for the combination of parameters of energy depletion (weight loss) and protein depletion (loss of fat free mass) as suggested in the ESPEN definition.

### Geriatric outpatient population

The prevalence of malnutrition according to the ESPEN definition in this study (8.2%) is in line with a recently published meta-analysis by Cereda et al. using the full Mini Nutritional Assessment (MNA) in geriatric outpatients (6.4%) [2]. Our study also confirms the previously reported associations between (the risk of) malnutrition (mostly defined by MNA) and HGS, SPPB score, TUG and HADS depression score [4–6, 13].

In the present study, a trend was observed for the association between being malnourished and falls. This is in discrepancy with a study of van Bokhorst de van der Schueren et al. [6] where falls were recalled differently as ever versus never and the present study reported a fall (yes/no) in the past 12 months.

In the present study, no association was found between being malnourished and a low MMSE score which is in line with previous studies [6, 14] in a population of geriatric patients.

In previous studies, the terms “malnutrition” and “risk of malnutrition” were often used interchangeably, leading to higher prevalence rates of malnutrition. The variety of settings in which studies were performed may also explain the different prevalence rates and different associations between malnutrition and outcome measures.

### Strengths and limitations

This study is the first to describe the association between the new ESPEN criteria for malnutrition and clinically relevant outcome measures. In addition, the clinically relevant outcome measures that were used were measured objectively, except for falls and HADS, which were obtained by questionnaires.

A limitation of this study is the small sample of malnourished outpatients. The low prevalence might be a definition problem: if the definition is too strict, the prevalence will always be low. Another problem might be that geriatric outpatients are often at risk for malnutrition instead of being diagnosed as malnourished; until now the ESPEN definition does not have a category for at-risk patients. Furthermore, the complexity of diagnosing malnutrition is a limitation; for example cognitive impairment, depression or falls may be risk factors as well as outcome factors. A final limitation is that a small inter-observer variability could have occurred, although standard operating procedures were applied.

## Conclusion

This study is the first to confirm the association between being malnourished, defined by the new ESPEN definition, and clinically relevant outcome measures in a population of geriatric outpatients. Future research should focus on determining the predictive value of the ESPEN definition and thereby taking the different cut off points used into account.
